# Listening to the Post-script: Intersections Between Improvisation and Indeterminacy in Music and Education

**DOI:** 10.1007/s42438-022-00326-z

**Published:** 2022-08-10

**Authors:** Stephanie Wilson

**Affiliations:** Business School, The University of Sydney, University of Sydney, Sydney, Australia

**Keywords:** Improvisation, Indeterminacy, Higher education, Soundscape, Timescape

## Abstract

This article begins by exploring how current educational research describes the role of improvisation in education. Drawing on Biesta’s (2017) discussion of the purpose of the educational task, it then argues for a broader consideration of the role of improvisation in education and its potential to positively disrupt traditional linear timescapes. While discussions of improvisation in education often reference jazz music, this study explores a broader range of indeterminate musical forms to gain different insights. Specifically, it translates Stern’s (1988) Table of Transformational and Musical Hierarchies into a framework for education to support educators in considering the role of improvisation in their design and teaching practice. The adapted conceptual model incorporates the dimensions of complex learning environments described by the Activity-Centred Analysis and Design (ACAD) framework as discussed by Goodyear and Carvalho (2014), and in the process, surfaces the temporal aspects of the ACAD framework for closer examination. The model highlights several factors that take on a more significant role in highly indeterminate educational forms such as silence, absence, openings, and rupture. It also provides an opportunity to reflect on ways in which technology contributes to enabling or constraining improvisational processes in postdigital education. The study contributes to research on the intersection between sound and education, and to making the sonic dimension of education one of more ‘intentional concern’ as described by Ford and Sasaki (2021).

## Introduction

Recent shifts to online learning caused by the Covid-19 pandemic have drawn attention to the role of improvisation in education, making it timely to revisit through research. As suggested by Boys (2022: 17), ‘the common sense assumptions and everyday routines that are the often unnoticed underpinning of teaching and learning practices are best revealed when these are breached’. In a study by Green (2022: 108), teacher-designers reported a ‘loss of spontaneous responses to perceived student needs’ in the move to online teaching. Citing Fawns (2019), Green (2022: 108) notes that teachers could ‘no longer use known resources and experience developed over years of teaching, which in the physical scenario enabled instant responses to student learning needs’. Similar challenges were experienced by music improvisation artists when attempting to move their practice online (Cai and Terry 2020). While teachers may not always be aware of their improvisational practices, their reliance on it was foregrounded when the online teaching environment limited their capacity to use it in their practice. While many felt that their ability to improvise was inhibited in the online environment, aspects of emergency remote teaching called for greater levels of improvisation, particularly in relation to the adaptation of teaching and learning spaces. As suggested by Gourlay (2020: 807), ‘the sudden and enforced nature of the lockdown necessitated this sort of creative improvisation, in which spaces which were hitherto private, domestic, and intimate are [changed] in their nature, arguably becoming outposts of the campus and the world of work’.

While the pandemic has brought some aspects of the role of improvisation in education to light, the increasingly complex and uncertain nature of higher education more generally has also been recognised as requiring teachers to possess a higher degree of improvisational ability. For example, taking a sociomaterial perspective, Pischetola (2022: 75) notes that ‘a large range of contexts, materials and practices’ gives rise to uncertainty, and citing Fenwick (2011), she reinforces that ‘the element of uncertainty is particularly relevant as the unfolding of multiple possibilities makes actions, choices and sense-making processes constantly unpredictable’ (Pischetola 2022: 75). Further, she cites Morin et al. (2003) to support the idea that ‘teachers should always be able to improvise or, in other words, to adapt to the non-linearity of a complex system’ (Pischetola 2022: 75). The partial return to on-campus teaching in many universities provides an important opportunity to revisit the purpose of the educational task, to reinforce what we value about our current approaches, and think carefully about which aspects of our educational practice should be retained and discarded. This includes a consideration of the balance of structure and improvisation in teaching and learning, and the extent to which technology enables or constrains improvisational processes in postdigital education.

The literature cited above suggests that improvisational practices may take on a heightened role in the postdigital educational environment. In his study of postdigital education in design and practice, Fawns (2019) reflects on research by Cuban (2001) and Enrique (2009) that examined how teachers use technology in their practice. These studies found that ‘teachers did not simply follow the expectations of either institution or technology designers in how they used technology’ but rather that ‘teaching practices, like any practices, involve workarounds, subversions and improvisations’ (Fawns 2019: 137). The studies revealed that while institutional expectations and policies shaped practices to some extent, ‘they are also situated and contextual, emerging out of a complex tapestry of conditions and parameters that cannot be predetermined’ (138).

To further understand these different perspectives on improvisation in education, and given the long history of improvisation in the field of music, it is appropriate to acknowledge studies that focus on the intersections between sound and education. Recent studies in this field have advocated for multimodal approaches to understanding education and have demonstrated how sound studies can help us rethink a wide range of educational concerns. For example, studies by Wargo et al. (2021) advocate a multimodal approach to educational ethnography that brings sound into equal importance with the visual and textual, and Ceraso (2018) demonstrates the benefits of using multimodal listening in her work on sonic pedagogy. Other examples include Elbow’s (2006) *The Music of Form*, which examines the organisation of music to rethink the organisation of writing, and Ahern’s (2022) recent research on the potential of designing soundscapes for learners in the online environment. Wilson (2022) considers how an understanding of spatial representations of form in music can inform how we might visualise the structure of learning sessions in the hybrid learning environment to support educational design and teaching.

Sound studies in education also highlight how our listening practices have evolved over time based on the increasing presence of technology in our listening environment. Petar Jandrić’s interview with electronic music composer Kim Cascone sheds light on how the use of electronics in music evolved over several decades into new forms of computer and digital music, continuing to transform our understanding and experience of sound (Cascone and Jandrić 2021). Cascone’s reflections prompt us to think about the extent to which we may want to foreground or background digital tools to achieve certain goals that we value in education. Recent research investigating the impact of the pandemic on improvisation in music clearly demonstrates that improvisation practiced by musicians who are co-located in the same physical environment is not the same as that practiced by musicians collaborating online (McDonald et al. 2021; Cai and Terry 2020). These studies highlight some of the key constraints of improvising online (such as latency and sound quality), but also how those constraints have led to new improvisational practices where technology has the potential to become another ‘player’ in the process. In the same way that we are coming to understand the limitations and benefits of practicing musical improvisation online, we need to better understand how technology might enable or inhibit improvisational practices in postdigital education.

Ford and Sasaki show how the ‘sounds and voices of digital technologies’ have led to new ‘posthuman practices of listening’ (2021: 116). Based on Schafer’s (1977) seminal work, *The Soundscape: Our Sonic Environment and the Tuning of the World*, they review the key transformations in soundscape studies that have led us to this point. One of these transformations was marked by the introduction of ‘aleatory processes where sounds are subjected to chance and contingency’ (1977: 116). This being the case, studies that help us understand the continuum from fixed structures to those approaching near total randomness in relation to musical composition have the potential to provide new ways of thinking about improvisation and indeterminacy in education, and support reflection on the structures and temporalities of postdigital education more broadly.

Within this context, this paper begins by exploring how current educational research describes the role of improvisation in education. Drawing on Biesta’s (2017) discussion of the purpose of the educational task, it then argues for a broader consideration of the role of improvisation in education and its potential to positively disrupt traditional linear timescapes. While discussions of improvisation in education often reference jazz music, this study explores a broader range of indeterminate musical forms to gain different insights. Specifically, it translates Stern’s (1988) Table of Transformational and Musical Hierarchies into a framework for education to support educators in considering the role of improvisation in their design and teaching practice. The adapted framework incorporates the dimensions of complex learning environments described by the Activity-Centred Analysis and Design (ACAD) framework (Goodyear and Carvalho 2014), and in the process, surfaces the temporal aspects of the ACAD framework for closer examination. The framework proposed in the present study highlights several factors that take on a more significant role in highly indeterminate educational forms such as silence, absence, openings, and rupture. The study contributes to research on the intersection between sound and education, and to making the sonic dimension of education one of more ‘intentional concern’ (Ford and Sasaki 2021).

## The Role of Improvisation in Education

This opening section considers the focus of research on improvisation in education. It is not intended as a comprehensive review of literature, but rather to identify some of the ways researchers have described the role improvisation plays in the educational process. Keith Sawyer’s (2011a) edited volume, *Structure and Improvisation in Creative Teaching*, brings together one of the more comprehensive collections of research investigating aspects of improvisation in education, and is therefore the primary focus here. Contributions include studies exploring: the relationship between expert teaching and teachers’ capacity to improvise; how to develop teachers’ improvisational skills in the classroom (for example, DeZutter 2011); and the use of ‘disciplined improvisation’ in teaching to encourage creativity (for example, Berghetto and Kauffman 2011; Burnard 2011; Perone 2011).

Based on the collection, Sawyer argues that spontaneity and improvisation in the classroom ‘requires no less skill than improvisation does in jazz, dance and theatre’ (2011: xvi). He highlights research that has demonstrated that experienced teachers are ‘better at improvising in response to each class’s unique flow’ and spend ‘less advance time planning than novice teachers’ (1). He also points out the paradox that while experienced teachers use more routines and activity structures, they also improvise more. Sawyer emphasises that the challenge for teachers is to find the balance between structure and improvisation to optimise student learning, describing this balance as ‘the essence of the art of teaching’ (2). In discussing the relationship between structure and agency in the classroom, he suggests that ‘constructivism is not a free-wheeling student-centred caricature’, but rather ‘constructivist learning proceeds more effectively in the presence of *scaffolds*’, that is, loose structures that are carefully designed to guide students as they improvise towards content, knowledge, skills, and deeper conceptual understanding (3).

In the introductory chapter, Sawyer (2011b) discusses research on improvisation in three key areas: teaching as performance, teacher expertise, and creative teaching and learning. When discussing teaching as performance he refers to work by Eisner (1979) who described teaching as an art in four ways: when students perceive the classroom to be ‘aesthetic’; when teachers ‘make judgements based largely on qualities that unfold during the course of action’ (the improvisational element of teaching); when teaching is not limited to routines, but is responsive to the ‘contingencies’ of each classroom; and when the ends achieved by teachers are ‘emergent … found in the course of interaction with students’ (Sawyer 2011b: 4). Sawyer cautions against some aspects of the ‘teaching as art’ metaphor (see also Sawyer 2004), particularly when there is an emphasis on teacher as ‘performer’, arguing that it can take attention away from the interactive elements of teaching and learning. Aligning with Borko and Livingston (1989), he focuses instead on *improvisational* performance, making comparisons with transmission models of teaching. He suggests that skilful improvisation ‘resides at the tension between structure and freedom’ and, along with Erickson (2011), reinforces that good improvisatory performance relies on structures and skills (Sawyer 2011b: 5).

In relation to research on teacher expertise, Sawyer (2011b) makes links with both Schön’s (1983) work on reflective practice, pointing out that his notion of what it means to be professional corresponds with ‘the ability to improvise effectively within structures’ (Sawyer 2011b: 7), and Shulman’s (1987) research on the ‘wisdom of practice’. Further, he refers to Eisner’s (1979) focus on the uncertainty of classrooms and the need for teachers to develop an ‘educational imagination’ that allows them to ‘balance structure and spontaneity’ (Sawyer 2011b: 7). Also referred to are studies on classroom discourse, a feature of teaching and learning that is often described as improvisational (see for example Mehan 1979; Erickson 1982). Sawyer cites Borko and Livingston (1989) who found that ‘expert teachers notice different aspects of classrooms than do novices, are more selective in their use of information during planning and interactive teaching, and make greater use of instructional and management routines’ (Sawyer 2011b: 8). Much of the research above on teacher expertise focuses on questions about knowledge structures and their relationship with the improvisational features of practice.

Sawyer (2011b) uses the term ‘disciplined improvisation’ to describe what teachers engage in when they apply their expertise (which includes the knowledge base of rules, plans, and structures they develop over time) in improvisational practice, and discusses research that has sought to understand how ‘the fixed structures of expertise become realised in the everyday improvisation’ of classroom practice (11). He highlights the parallel misconception in jazz and classroom improvisation that improvisation means ‘anything goes’, reminding us that jazz improvisation requires ‘a deep knowledge of complex harmonic structures and a profound familiarity with the large body of *standards*…’ (12). In other words, both teaching and jazz improvisation require a balance between structure and creativity. Further studies make comparisons with improvisational practices in other disciplines such as theatre (Shem-Tov 2011) and dance choreography (Fournier 2011).

In describing how improvisation supports creativity, Sawyer (2011b) refers to research on both the creativity of teachers, and the kinds of learning environments that promote creativity in students. He emphasises that the capacities identified in creative teaching and learning align with valued twenty-first century skills associated with creativity and innovation.

In summary, research on improvisation in education tends to centre around the relationship between expert teaching and teachers’ capacity to improvise, approaches to developing teachers’ improvisational skills, and the use of disciplined improvisation in teaching to encourage creativity. What the studies cited above don’t explicitly address is the role of technology in teachers’ and learners’ improvisatory practices, how it enables or hinders their ability to improvise, or how it contributes to determining activity. In postdigital education, we need to better understand the constraints, possibilities and risk of technology (Fawns 2022). In terms of improvisation, this means gaining a better understanding of ways in which technology might enable or constrain improvisational processes and outcomes.

A dominant perspective in the educational literature is that when teachers become skilled at improvisational practice, their students learn more effectively, and that improvisation supports constructivism. However, it is important to consider whether there may be reasons for incorporating improvisational practices in education that go beyond the promotion of student-centred learning. In presenting a model of entangled pedagogy, Fawns (2022) foregrounds the importance of making purpose, context and values explicit, and regularly revisiting them ‘to ensure that they meaningfully and iteratively inform choices around methods and technology, whilst also recognising the shaping role of technology and methods as part of the pedagogical mix’ (9).

## Indeterminacy, Improvisation, and the Educational Task

This section reviews Biesta’s (2017) discussion of the educational task, to help us consider whether there might be additional roles for improvisation in education that go beyond supporting student-centred learning. In his book, *The Rediscovery of Teaching* (Biesta 2017), he cautions against recent conceptions of teaching that see the main role of the teacher as the ‘the guide on the side’. In re-examining the role of the teacher, he revisits fundamental questions about the purpose of education. In his view, the purpose of the educational task is ‘arousing the desire in another human being for wanting to exist in and with the world in a grown-up way, that is as *subject*’ (Biesta 2017: 7). To exist as subject is to be in a ‘state of dialogue with who and what is other’ (4). Similarly, a grown-up way of existing acknowledges the ‘alterity and integrity of what and who is other’, and this includes existing ‘in the world without considering oneself as the centre, origin or ground of the world…’ (8).

Central to the concept of grown-up-ness is how we respond when we encounter resistance. That is, when someone or something resists our initiatives (Biesta 2017: 14). This encounter is considered particularly important, as it ‘shows that the world is not a construction of our mind or our desires, but actually has an existence and hence integrity of its own’ (14). Biesta describes two extremes in terms of how one might respond when encountering resistance to one’s initiatives. The first is to ‘blame’ that which offers resistance, leading us to enforce our intentions. If we take this too far it can ‘destroy the (integrity of the) very *entity* that offers resistance’ (14). Biesta describes this extreme as *world destruction*. At the other extreme, is to ‘withdraw from what offers resistance, to step away from it’, because it is seen as too complex or difficult. In this sense, at the extreme, we may ‘withdraw ourselves completely from (existence in) the world’ (14). Biesta refers to this as *self-destruction.* In the middle ground between the two extremes is where existence in and with the world takes place. Biesta refers to this middle ground as *dialogue*—‘a way of being together that seeks to do justice to all partners involved’ (14). While Biesta acknowledges that there are times when we need to retreat from the middle ground, or push for something better, the middle ground should be embraced as it is the thing that makes our existence possible. It requires ‘a desire for worldly existence, an existence outside of ourselves’, and Biesta argues that the educational task involves ‘arousing such a desire in another human being’ (15).

What role might indeterminacy and improvisation play in helping us support students in staying in the middle ground, in dialogue with ‘the other’? If the middle ground is ‘a place where our self-expression encounters limits, interruptions, responses…’ (Biesta 2017: 17) then how might we, as educational designers and teachers, use improvisation and indeterminacy to generate environments that enable students to encounter these factors? And what do we need to know about the interplay between technology, tasks and social interactions to best support such practices? Biesta describes three possible ways that teachers can help students exist in the world in a grown-up way. These include interruption, suspension and sustenance. Interruption is concerned with the question of ‘which development is desirable and which is not’, thereby interrupting and questioning development (17). Suspension refers to suspension in time and space that provides an opportunity to establish ‘relationships with our desires, to make them visible, perceivable, so that we can work on them’, selecting and transforming them (18). Interruption and suspension both exist in the middle ground. Finally, sustenance involves the work of helping students stay in the sometimes difficult and challenging middle ground (19). In this sense, the educational task involves ‘giving form to the experience of resistance’ through pedagogy and curriculum. This paper considers the role of improvisation and indeterminacy as one way to stage ‘the experience of resistance as important, meaningful and positive…’ (19). Listening and silence, which play a key role in improvisatory processes, are also important in the process of encountering resistance and contributing to a grown up way of existing that acknowledges ‘who and what is other’ (4). For example, silence can be used to enable deep listening practices (Goodyear 2022) and to provide opportunities to ‘listen to our listening’ (Ford and Sasaki 2021). Ceraso (2018) suggests that teachers and students ‘regard sound as a locus of inquiry … embrace experimentation and unfamiliar sonic practices’ and notes that ‘multimodal listening allows students to figuratively and literally make sense of their own and others’ lived experiences’ (12, 154).

Biesta (2017) offers an understanding of grown-up-ness ‘that doesn’t see it as the outcome of a developmental or educational trajectory but rather as a way of existing in and with the world, a way where the question of whether what we desire is what we should be desiring has become a living question, a question we carry with us and bring into play in every situation we encounter’ (4). By questioning the idea that grown-up-ness, the proposed aim of the educational task, is not brought about by a linear developmental process, Biesta is questioning dominant conceptions of time in education. By doing so, he is also questioning whether there is more to the educational task than learning, which is centred around comprehension and sense-making and involves changes in cognition, understanding, and skill, for example (86). Biesta’s questioning of current conceptions of temporality in education invites an examination of the role played by indeterminacy and improvisation in shaping the temporal landscape; and further, how technology might contribute to new temporalities in education through the complex interplay between people, tasks, and environment.

If there is value in interrupting the logic tied to a conception of teaching associated with student’s growth (and thereby challenging dominant notions of time in education), then could indeterminate compositions (educational designs) that often result in more non-linear forms be one way to achieve this? How might a deeper look at the role of improvisation and indeterminacy help us to move away from linear conceptions of time to more cyclic conceptions of time (Biesta 2017; Ford 2022)? Biesta (2017) questions whether a conception of time in education concerned with development over time can ‘fully capture education’s interest in freedom’ and proposes a *non-temporal* educational logic to combat this (86). In this paper, I argue that if the educational task is about helping students stay in the middle ground, and if non-temporal or at least non-linear forms are needed to challenge ways that ‘educational processes and practices are being understood, enacted, theorised and researched’ (86), then an exploration of educational forms that range from ‘as fixed as possible’ to ‘near total randomness’ may be helpful (Stern 1988: 104).

Currently in education there are no frameworks available that explicitly identify, analyse, and evaluate indeterminate elements and procedures. Such frameworks would be useful in helping us understand the continuum between structurally defined and structurally undefined educational forms, and to understand more fully what the aleatoric educational environment looks like. The purpose would be to provide practical resources that would allow us to be more deliberate in the choices we make as educational designers and teachers to achieve the educational goals we see as important, particularly if we want to ‘orient our actions towards that which is not visible in the here and now – the student’s subject-ness’ and to ‘close our eyes to what is visible, to the *evidence* that tries to tell us that the student is not yet ready…’ (Biesta 2017: 94). As part of the process, we need to need to better understand and recognise the ‘shaping role of technology and methods’ and how they feature in the pedagogical mix (Fawns 2022), particularly as we work with less structurally defined educational forms.

## The Aleatoric Soundscape: Indeterminacy and Improvisation in Music

A musical composition or performance may contain no improvisation at all, with every note and expressive element tightly determined by musical notation, or conversely, it might be completely improvised, with nothing predetermined. The musical score often dictates the level of improvisation intended for a piece of music and can be likened to a teacher’s *script* for a lesson—very detailed scripts may suggest little room for improvisation and flexibility in the teaching and learning approaches taken, while a more *skeletal* plan might suggest room for a more improvisational approach where the student (like the performer in a musical sense) has more of a role to play.

As mentioned previously, comparisons between improvisation in music and education in the literature tend to prioritise jazz improvisation. While this provides useful insights into understanding the relationship between structure and agency in education, other forms of improvisation in music (and their associated compositional processes) may be useful in helping us to further consider how our educational designs can be structured to include improvisatory elements, and to revisit the question of how this might support the kinds of educational experiences we value.

This section begins with a brief description of indeterminacy in twentieth-century Western art music, defining key associated terms, such as aleatory, random, and chance music, and acknowledging the important role of technology in generating new musical structures. It then introduces a framework proposed by Stern (1988) that is later translated for the educational context.

### Indeterminacy and Related Terms

Indeterminacy in music refers to an approach to composition where some of the aspects of the work are left open to chance or to the discretion of the performer. Composers of such works often used graphic notation and text instructions in addition to traditional musical notation. American composer Charles Ives, who left some aspects of his compositions open to the performer, is considered one of the pioneers of indeterminacy in the early twentieth century. In the 1930s, composers such as Henry Cowell extended the idea of performer freedom, for example by creating works that allowed performers to arrange musical fragments into various sequences, and by experimenting with *elastic form*. However, indeterminate composition is most commonly associated with composers of the New York School such as John Cage, Earl Browne, Morton Feldman, and Christian Wolff in the 1950s, as well as European composers such as Pierre Boulez, Karlheinz Stockhausen, and Witold Lutoslowski.

In music, the terms indeterminacy, chance, and random or aleatory music are often used interchangeably to describe different levels or types of control over the elements of a composition. As suggested by Stern (1988), each of these terms suggests ‘a relinquishment over the final, definitive arrangement of the musical materials’ (103). In music, it is useful to differentiate these terms to examine their relationship to improvisation.

Chance music implies that the composer relinquishes control at some stage of the *compositional* process (a foundational example being John Cage’s *Music of Changes*). In education, the equivalent would involve the teacher-designer relinquishing control at some point in the *design* process. Often chance music is precise, in that it leaves little room for performer freedom (the compositional process uses chance as a device, but the composition is then *set* accordingly). In indeterminate composition, however, the unpredictable elements occur during *performance*. Such compositions demand a greater contribution by the performer(s) to the final arrangement of musical material (Griffiths 1980). The definition of aleatory can be extended to incorporate both indeterminacy and chance music, that is, it is ‘a term applied to music whose composition and/or performance is, to a greater or lesser extent, undetermined by the composer’ (237). While indeterminate music and improvised music share many similarities, the literature on contemporary indeterminate music tends to avoid using the term improvisation, and likewise, the literature on improvisation makes reference to contemporary indeterminate music only infrequently. This may account for why studies on improvisation in education tend to focus on jazz music as a comparison.

The composer Earl Brown suggests that improvisation and chance are opposed concepts, because chance music involves removing the element of choice, and consequently taste, at some stage in the compositional process. He refers to John Cage’s use of chance in which he flipped coins to determine which sound event came next in a sequence (removing *choice*). In this case, chance was used to *fix* musical elements, and the musicians were not offered an element of choice (Bailey 1992). While chance and improvisation might be seen as opposed concepts, indeterminacy and improvisation can be seen as more closely related. The difference may be that while improvisation is a necessary prerequisite for the realisation of indeterminate music, improvisation is not dependent on indeterminate composition for its existence. Improvisation can exist without the guidance of an *incomplete* score. Therefore, while indeterminate composition demands that an improvisation be shaped according to the elements present in a score (or model), *free* improvisation is guided only by the *invisible* model defined by the memory and the experience of the performers involved.

As suggested by Stern (1988), improvisation is a performer’s term, and ‘its use as an independent variable in aleatoric composition bespeaks a new relationship between performer and composer’ (108). The influence of the composer on the indeterminate elements of a composition must be considered in the analysis of such compositions. The question of whether ‘any’ realisation of an indeterminate work is accepted by the composer is a further consideration. Some composers work towards an *ideal* performance, while others may literally accept any result. Ferand (1961) attributed the prevalence of improvisation early in music’s history to the fact that ‘inventor and performer of a tune were usually the same person’ (5). Sometimes composers gradually *fix* their compositions over time. That is, pieces of music that begin as free improvisations (based on very *open* scores) may end up as something that could be described as *written out* improvisations. For example, John Cage describes ‘Feldman’s conventionally notated music [as] himself playing his graph music’ (Nyman 1974: 45).

Another useful distinction to make is that between improvisation and interpretation. Many would argue that all performers improvise to some extent. That is, it is not possible for a composer to communicate every detail of intention via a score; therefore, the interpretation of the score becomes the responsibility of the performer. Indeterminate composition however involves a deliberate choice by the composer to allow for greater performer contribution (Wilson 1994).

### Organising Procedures Involving Indeterminacy and Improvisation

In a paper published in 1988, Max Stern, composer, musician, conductor, and critic, explored organising procedures involving indeterminacy and improvisation in music. He proposed a ‘conceptual model for identifying, analysing, and evaluating indeterminate elements and procedures used in composition’ (Stern 1988: 108). The model, which allows us to assess compositions on a continuum from ‘as fixed as possible’ to ‘near total randomness’ is explored here as a foundation for considering synergies with organising procedures in education. Technology has had a profound influence on the emergence of non-linear forms of music, and this called for new analytical approaches to help us understand and describe temporality in contemporary music. It follows that similar frameworks in education may be needed to better accommodate a broader range of structures and temporalities that are emerging as a result of the complex entanglement of people, tasks, and technology.

While improvisation has been a feature of music through different periods of music history, it became less of a feature with the introduction of Western tonal music (from 1650 to 1900) that was organised functionally through tonality. In the twentieth century, however, we began to see a radical departure from traditional musical forms in terms of tonality, temporal characteristics, and compositional approaches, some of which were greatly influenced by new technologies. Along with indeterminate music, these departures included the introduction of compositional methods such as the *twelve-tone system* and *serial music*. In these new forms, tone and other musical parameters were no longer tied to function. That is, they could exist on their own terms. Stern uses the term ‘denotative’ to describe this new way of seeing music as ‘parameters’. This is contrasted with music that is relational and associative, which is referred to as connotative (where there is an interrelationship between all the elements of music).

Stern (1988) suggests that the ‘aleatoric sound image’ that resulted from this release from a relational structure is easier to define by what is absent, rather than by what is present (108). For example, aleatoric soundscapes include characteristics such as:

1. Avoidance of all traditional symmetry and continuity.
2. Athematic, with no attempt at motivic organization.
3. Arhythmic, with no sense of pulsation.
4. Events that are highly irregular and unpredictable.
5. Tension flow lacking.
6. Separate dimensions added together and not fused. (Stern 1988: 108)

**Fig. 1:**
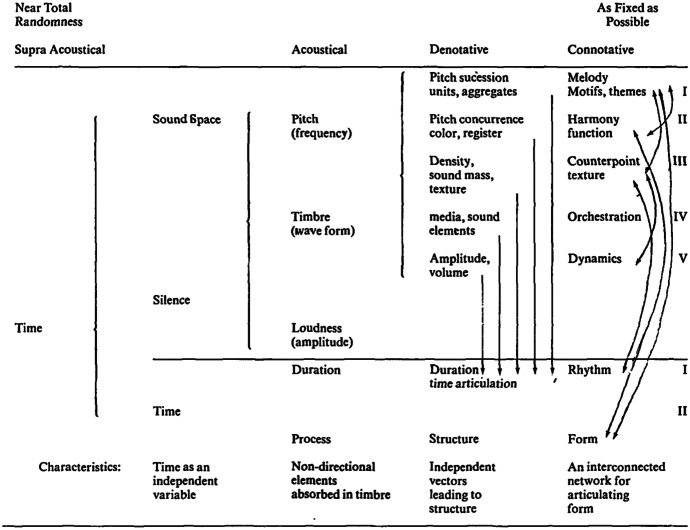
Table of Transformational and Musical Hierarchies (Stern 1988: 104)

To accommodate aleatoric music, Stern (1988: 104) presents a Table of Musical and Transformational Hierarchies that arranges the connotative-denotative dichotomy into a continuum (Fig. 1). The horizontal continuum represents *time*, ranging from ‘abstract time, through various levels of durational processes, to discrete durations and time-articulating structures, which eventually evolve into coherent forms’ (Stern 1988: 105). Stern describes *sound space* as the ‘total sound-silence material which is to be shaped’ (109), and it can include ‘events which occur at any given point in time’ (105). In relation to the *supra-acoustical* and *acoustical regions* of the model, Stern points out that they may also ‘imply pre-conscious, sub-conscious, or pre-composition dimensions of the creative process with respect to the denotative-connotative region’ (Stern 1988: 105).

The straight arrows in the denotative region indicate ‘that in denotative structures all parameters of sound may relate to the overall structure as discrete elements’, while the curved arrows in the connotative region indicate that in connotative structures there is an interrelationship between the musical elements such as melody, harmony, and rhythm (Stern 1988: 105).

Focusing on one dimension of the model, pitch (frequency), we can see that it is broken down into several parameters in the denotative region, the first being pitch succession units, and aggregates (where succession does not imply form). If the intention of the composer is to articulate form, then these elements of pitch would need to be organised into melodies, motifs, or themes. Similarly, pitch concurrence (the second parameter of pitch) on its own does not imply form, but can be given form by organising pitch concurrence in accordance with harmonic structures which may then have a function in the overall movement of the musical work when combined with other parameters. The remaining parameters in the acoustical region of the model are presented in a similar way, specifying denotative and connotative elements.

## Towards a Model of Indeterminacy and Improvisation in Education

**Fig. 2:**
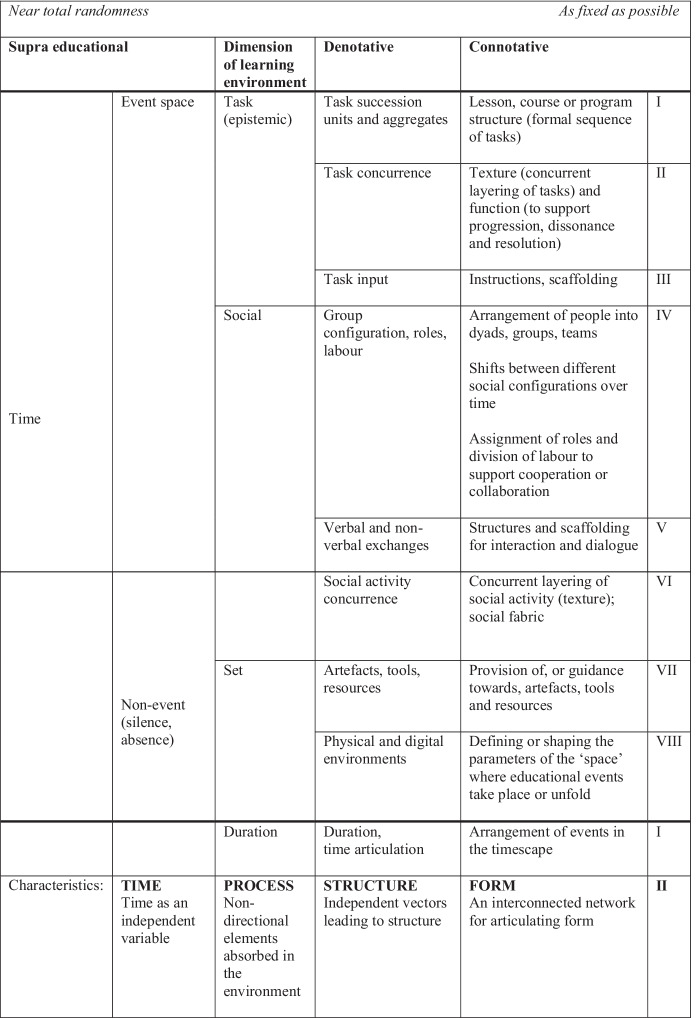
An adaptation of Stern’s (1988: 104) model as applied to education, incorporating the dimensions of the ACAD framework (Goodyear and Carvalho 2014)

In this section, Stern’s (1988) model is translated into the educational context to consider its usefulness in helping us identify, analyse, and evaluate indeterminate elements and procedures in education, and to better understand the complex entanglements between people, tasks, and technology and how they influence educational structures (Fig. 2). In this model, *sound space* is replaced by the educational *event space*, and silence is broadened to the category of *non-event* (which may include silence or absence). The *acoustical* dimensions in Stern’s model are replaced by the three broad dimensions of the Activity-Centred Analysis and Design (ACAD) framework (Goodyear and Carvalho 2014). These include task (epistemic), social, and set. In the ACAD framework, epistemic design involves a consideration of tasks; social design includes how learners are grouped and the roles and responsibilities we assign to them; and set design refers to the physical and virtual learning and teaching environment and includes tools, artefact and resources.

The breakdown of the pitch dimension into denotative and connotative elements in Stern’s (1988) model provides a new way of thinking about the task dimension. At the denotative level, task can be broken down into task succession units and aggregates, task concurrence, and task input. When we want to articulate form, task succession units and aggregates can be shaped to become lessons, courses, and programs. Similarly, while tasks occurring simultaneously may have no relationship to one another (acting as discrete elements), we can articulate form by giving the layering of tasks a particular function, such as progression, dissonance or resolution. Similarly, while task input on its own does not imply form, the addition of task instructions and scaffolding add to the interconnected network for articulating form. Rather than including curved arrows to indicate interrelations (as Stern has done), the model assumes that connotative structures may involve interrelationships between any or all of the elements in the connotative region.

**Fig. 3:**
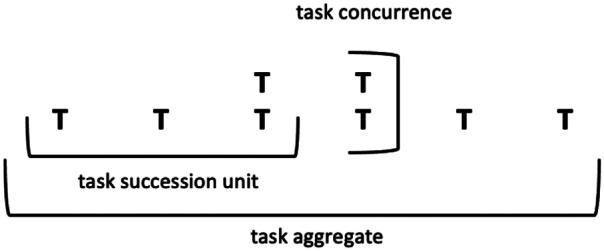
Denotative aspects associated with the task dimension

Figure 3 further illustrates some of the denotative aspects of the task dimension. As suggested above, tasks may appear in succession units, concurrently, or as an aggregate, but it is their interconnectedness with other educational elements that gives them form. Educational designs may choose to build more or less form into the task dimension and combine it with other dimensions and elements in various ways to achieve either very defined structures, or structures that move towards the random end of the continuum.

If we consider the social dimension, the denotative region includes three main elements: group configuration, roles, and labour (as specified by the ACAD framework), but also verbal and non-verbal exchanges, and social activity concurrence. While group configuration is a parameter on its own, it only contributes to form when combined with other educational elements. In our educational designs we may choose to provide different degrees of scaffolding for verbal and non-verbal exchanges to support social interaction, and similarly we may deliberately alternate between different social configurations over time to support progression towards a goal, for example if we want to encourage the cross-fertilisation of ideas between groups of different configurations. Social activity can also be layered, with different types of social activity and interaction occurring at the same time. Again, all of these social elements can be carefully designed or be left to open.

The final dimension, set, includes artefacts, tools and resources, and physical and digital environments. As designers, we can choose to provide or guide students towards particular artefacts, tools, and resources to support tasks and social activity, or leave these elements open to aspects of improvisation. Similarly, we can shape physical and digital environments in particular ways to contribute to form, or deliberately leave these aspects undefined. Recent research examining differences between musicians’ experiences of improvising in person and online revealed that one of the greatest limitations to their improvisatory practice in the online environment was the inability to move quickly and fluidly between group configurations of different sizes (Cai and Terry 2020). This taken for granted aspect of music making, used to create dynamics in an improvised work, became difficult to achieve for the musicians interviewed in the study. In considering more indeterminate educational structures using the model, it is important to take into account ways in which technology might constrain or support improvisational elements when various parameters are combined (in this case, shifts between different social configurations over time, and the educational environment).

It is important to note here that *undefined* does not mean *unconsidered*, but rather the lack of definition or prescription is intentional to invite improvisation into the process (building indeterminacy into the design). The model presented above is intended to help us think about presence and absence in a more explicit way, and the extent to which educational designs promote linear and non-linear temporal forms. In educational design we often think about what to include, giving less attention to what to leave out. In adapting the durational and rhythmic elements of Stern’s (1988) model for education, we can begin to think about designs that range from highly linear forms through to ‘arrythmia’ (Ford 2022). I would argue that educational forms that have higher degrees of indeterminacy may also provide opportunities for teachers to practice the elements of interruption, suspension and sustenance proposed by Biesta (2017), thereby helping students to stay in the ‘middle ground’.

As an extension of Stern’s (1988) model, he identified a continuum of the various types and degrees of indeterminacy in relation to form-process, ranging from ‘completely composed to unstructured musical activity’ (as aleatoric compositions can be structurally defined or structurally undefined or anywhere in between) (108). These types included defined structural frameworks, a constructivist approach (not necessarily intended as a link to constructivism in a learning context), a translational equivalent, an open or mobile approach, a transformational procedure, and non-method. Table 1 shows how these different types or degrees of indeterminacy might be translated in the educational context in relation to the design of a single lesson (however the types could also be applied to a whole unit, course, or degree program).

**Table 1:** An adaptation of Stern’s degrees or levels of indeterminacy (Stern 1988: 108)

**Degree or level of indeterminacy**	**Description**
Defined structural frameworks	The structural framework of the lesson is completely designed (rather than being left to chance elements), but indeterminacy is incorporated into the design to varying degrees or for varying lengths (‘limited aleatorism’).
A ‘constructivist’ approach	The teacher determines the distribution of tasks, but the specifics are left to the students.
A translational equivalent	A transformational procedure whereby a source not traditionally used in education is used as the basis for generating the educational design. Once the designer has made the correspondence-relation decision, the design process tends to operate automatically. Depending on the source and the rules of the game established, the outcome can vary from near total control to a high degree of randomness.
An open or mobile approach	The components of the educational design (such as tasks, resources, and social configurations) are fixed, but the ordering of the components is left to students. The result is an unknown number of possible realisations.
A transformation procedure	Similar to a translational equivalent, but the non-educational source used to generate the design (for example the tasks, group configuration, and resources) is in itself indeterminate (e.g. cards).
Non-method	Tasks, ways of interacting with others, and the use of space, artefacts, tools, and resources is undefined. Verbal, graphic, or pictorial representation may be used to allow chance or random activity to generate the shape of the lesson and its content.

Stern’s (1988) description of organising techniques and procedures in relation to time in music can help us think about the temporal aspects of education, and how we might influence them through our designs (108). As in musical compositions, time and duration operate on different levels simultaneously in education. As shown by Wilson (2022), these levels and their interrelationships become particularly complex in hybrid learning environments when some students are learning on campus and others remotely, and with various combinations of synchronous and asynchronous modes of learning. In relation to a single lesson, this would include the overall length of a lesson, proportions of parts or sections, and durations or individual particulars. The time frame of a lesson can be subjectively derived (for example from educational materials, or from working through a form-generating process), or objectively determined. In music, time frames might be objectively determined by random choice, a chance procedure, a numerical manipulation, or a natural event (Stern 1988: 108). Structure and form-generating processes can be used to generate a time frame or alternatively be placed within the time frame (108). Fawns (2019) reminds us that ‘learning is not only socially and materially distributed, but also temporally’ (141). By incorporating the dimensions of the ACAD framework into the model, it is hoped that this also serves to further foreground the temporal dimensions of the ACAD framework.

Table 2 provides some examples of design options for each of the dimensions of the ACAD framework. Specifically, and drawing on Stern (1988) once again, these move from determined, to relatively determined, to relatively undetermined, to non-determined. In relation to the model, the more undefined elements we incorporate into our designs, the more we move through the continuum towards near total randomness.

**Table 2:** Examples of educational design options based on the dimensions of the ACAD framework (Goodyear and Carvalho 2014) and arranged using Stern’s (1988) levels of indeterminacy

**ACAD dimension**	**Examples moving from determined, to relatively determined, to relatively undetermined, to non-determined**
Task design	1. Tasks are defined, and run in a pre-determined order. 2. Students are given a number of set tasks but can choose the order in which to engage with them (open or mobile approach). 3. Students are given set tasks but are asked to choose a set number from within a range of choices to be completed over the time frame. 4. Students are given a task, but choose some of the associated parameters, e.g. the topic, the framework. 5. Students are given a provocation such as an image, an object, a poem, a piece of music (something different to a standard reading) to which they need to respond in relation to a topic, concept, theory etc. (that is, they are given a starting point but they need to find their own direction). 6. Students are given an end point and asked to find their way there; to determine their own path that leads to a common end point. 7. Students are given something to do, investigate, inquire about, but not given any specific instructions. 8. Students are asked to ‘adopt’ a concept. They are not expected to ‘learn’ anything about it, but to ‘sit with it’, ‘live with it’ and see how it ‘addresses them’ over a set time period (Biesta 2017: 34). 9. Students are asked to come up with the topics to be discussed (around a theme or not), and form discussion groups based on areas of interest.
Social design	1. Students are put into pre-defined dyads, groups, or teams. 2. Students choose their own group or team with a suggested number of students. 3. Students choose who they want to work with (group size undetermined). 4. Students given an organising device to form groups, e.g. number out of a hat. 5. Students given a specific role to play. 6. Students choose a role to play. 7. Student’s role left to chance.
Set design	1. Permanent and non-permanent objects in the learning environment are pre-determined. 2. Students choose from a set of artefacts, resources, tools to support their task/project etc. 3. Students configure their own space to suit their needs (studio-model). 4. Students bring in and choose freely what resources and tools to use: no limitations.

## Indeterminacy, Non-linearity, and Postdigital Education

When we consider the aleatoric landscape of education, which includes different degrees of indeterminacy and non-linearity, there are a number of themes that take on new significance. Among them are silence, absence, openings, and rupture. The following discussion briefly explores these themes, which also have resonance in the postdigital educational literature.

### Silence, Absence, and the Void

It’s not that one listens to or for silence, it’s that silence is the condition of every listening. (Grebowicz 2017)

You were once a citizen of a country called I Don’t Know… Remember the burning boat that took you there? Climb in. (Howe 2021)

Elements such as silence, absence, and the void in improvisation and indeterminate forms of music are explored briefly here to further consider their role in education. As will be demonstrated, they are also themes that have received renewed attention in the literature in relation to postdigital education.

Musicians who improvise often speak about the importance of silence. For example, Thelonious Monk, jazz pianist and composer, stated that ‘[w]hat you don’t play can be more important than what you do’ (in Remes 2020: 26). And there is no question that silence features heavily in the philosophy and work of composers working with indeterminacy. An often cited example is John Cage’s work *4′33’*’ which involves a performer sitting at a piano and opening the lid, then staying silent for the specified duration. The sounds of the work are created by the environment and the audience themselves, leading Cage to state that ‘there is no such thing as silence’ (Cage 1961: 51). Even well before Cage and his contemporaries, composers were experimenting with silence in new ways. As early as 1897, Allais produced a ‘noteless musical score’ that requires musicians to do no more than count the bars. In terms of tempo, the instruction simply states ‘slowly, jesting’. As Remes himself jests, ‘one should never play nothing too fast’ (Remes 2020: 12). Another early example includes Erwin Schulhoff’s *Five Picturesques* where the third movement of the five-movement piano piece titled *In Futurum* is completely silent (lasting 2 min, much longer than a traditional ‘rest’ in music), with the instruction for the musician to play ‘with as much expression and feeling as you like’ (12). In reviewing silence in music, Remes concludes that to the careful listener, ‘silence is just as diverse and multifaceted as any other phenomenon – sonic or otherwise’ (Remes 2020: 14).

While literature and film are not the main focus here, obvious examples of literary lacunae include Beckett’s *Waiting for Godot* and *Act Without Words*, and Gnedov’s poem which simply includes the poem’s title *Poem of the End* and a blank page. In his exploration of absence in cinema, Remes (2020) provides cinematic examples containing structured absences that ‘deprive spectators of images and sounds that seem like they *should* be there’ (19), suggesting the important role expectation plays: ‘expectation is central to the phenomenology of absence’ (Remes 2020: 4). Using works from the film maker Stan Brakhage as examples, Remes considers the impact of films without sound, such as in the film *Window Water Baby Moving* where there is no soundtrack. It is argued that the absence of a soundtrack is more powerful because it allows the viewer to hear the ‘silently audible rhythm[s] in cinema’ or experience the ‘musicality of vision’ (Remes 2020: 61). These ideas connect with Lefebvre’s (2013) rhythmanalysis, a method for analysing the rhythms of the everyday—where rhythms may be audible, but not necessarily so.

For architects and urban designers, considerations of empty space and voids are central, with Christopher Alexander offering specific guidance on how to create positive empty space and voids that contribute to a sense of wholeness. Central to Alexander’s notion of positive space is the idea that ‘every placement is also a displacement’, and in relation to the void, that ‘there must be a balance of calm and emptiness within the delirious detail’ (Alexander 2002: 255). A study by Yeoman and Wilson (2019) demonstrates how an empty space incorporated into the design of a learning space contributed to valued learning activity.

Not surprisingly, educators usually focus on what is present in education. However, a consideration of what is absent—‘omissions, erasures and silence’ (Remes 2020: 24), and expanding our perspective on education to encompass what is visible and invisible, audible and inaudible, can lead to different insights. In considering that ‘only by imagining how one might respond to the presence of these elements can one truly appreciate the effect of their absence’ (26), and that ‘absences are relative … [they] draw their identity from their relata’ (10), what might absence look like in education? That is, how might an understanding of absence help us create meaningful structured absences in our educational designs, potentially using or allowing improvisational and indeterminate elements and opportunities? Some researchers have recently addressed aspects of presence and absence in the context of postdigital education. For example, citing Law and Mol (2001), Gourlay (2022) describes synchronous online teaching as occurring in a fire space, which consists of ‘a flickering relation between absence and presence’ (63).

In film, the terms *pseudopresence* and *interstitial presence* have been used to describe cinematic presence. An actor is present in their films, but these presences are ‘intermediaries between actual physical presence and absence’ (Remes 2020: 19). These notions are less well understood in education; however, there are some recent studies that have explored the impact of presence in relation to hybrid learning. For example, Raes (2022) investigated differences between physical and remote presence in relation to both conceptual understanding and affective engagement (finding significant differences in experience of the latter in favour of physical presence).

Notions of presence and absence lead us back to Biesta’s proposal that as part of the educational task we need to make the visible invisible, and the invisible visible. Can structured absences, brought about by improvisation, indeterminacy or other means help students to surface ‘what seemed impossible’ (Biesta 2017: 83)? In what situations might ‘not knowing’ and the absence of certainty, meaning-making and signification be a positive thing? What implications do these ideas of silence, presence, absence, and voids have for us in education, and how might we create structured silences to support our educational goals? Finally, what would make those silences meaningful, keeping in mind the potential negative impacts associated with silencing learning spaces, particularly in cases where the overall preoccupation is to reduce noise (Ahern 2022). It might be more productive to think of silence not as a lack of noise but as an opportunity for pauses, and for deep listening practices (Goodyear 2022), and to create opportunities to ‘listen to our listening’ (Ford and Sasaki 2021). When considering the role of technology in contributing to productive silence, we can reflect on what happened when improvising musicians attempted to continue their practice online during lockdown. Initially, they saw the auditory latency caused by the technology as debilitating. However, over time they found new ways to get around it by using longer pauses and silences in their improvisations (turning latency into an emergent feature). They reported that this new aspect of their practice actually led to deeper listening: ‘For some, these extreme circumstances have forced them to listen more attentively to each other while playing together, a skill that is fundamental to social improvisation’ (Cai and Terry 2020: 3).

### Openings, Ruptures, and Strange Loops

Kairos… does not submit to chronos… [it] exists as a potential, a mode of improvisation, of responding to a sudden opening in the fabric of time. No theory can enable or plan for it… (Lohrey 2020: 255)

If we see improvisation and indeterminacy as processes that help create *openings* in the learning landscape, then when do we want these to occur, and more importantly why? It could be that we want to create openings to bring students into the process of creating new knowledge and original work. Or it may be because we want to challenge the linear conceptions of time that dominate our classrooms and find ways to deliberately rupture and hold spaces open, for example by introducing arrhythmia (Ford 2022), or through the process of negation, which ‘keeps open the gap between the world and signification, between meaning and sense’ and ‘operates by keeping sense indeterminate to meaning and signification’ (Ford 2019: 110). Or we might see openings as opportunities for the invisible to become visible (Biesta 2017). Remes (2020) also recognises the relationship between absence and openness by describing absence ‘not as a mere lack but as an experience of radical openness’ (28).

Some researchers have identified important reasons why we may want to challenge the conventional timescapes of higher education, including to provide a more equitable experience for students (see Bennett and Bourke 2017; Boys 2022). Openings might also be seen as being caused by ‘edge boundaries’ or glitches as discussed by Cascone and Jandrić (2021) in their discussion of nascent technologies. Rather than being seen as malfunction, failure is described by Cascone as the ‘a lack of intersection between intention and expectation’ (Cascone and Jandrić 2021: 569). To what extent can improvisation and indeterminacy encourage this kind of productive failure and facilitate the opening up of learning and experience? McDonald et al. (2021) provide examples of how improvising musicians made new aesthetic discoveries in their improvisational practice in the online environment during lockdown, where virtual backgrounds, the ‘theatre of the home’ and the ‘Zoomesphere’ became integral parts of the improvised work. In education, we might identify ways in which technology provides similar opportunities, to help us see ‘the data hidden in our perceptual blind spot… if we choose to shift our focus there…’ (Cascone 2000a: 13).

Biesta (2017) argues for the importance of ‘disarmament’, suggesting that ‘the arrival of subject-ness is precisely not the outcome of a developmental trajectory, is not the culmination of a learning trajectory, but an event that breaks through all this…’ (91). Central to this idea is the assumption of the equality of intelligence and the role of trust in educational relationships.

In music, composers’ experiments with non-linearity could be seen as attempts to create openings or ruptures in the temporal and sonic landscape. Approaches have included creating extremely dense musical textures, randomly ordering tones in a sequence, temporally displacing a melody through the technique of ‘phasing’, pervasive discontinuity, creating streams of unrelated sounds (e.g. using extreme timbral variation) and by generating very sparsely saturated sound fields. Examples of these forms may be fixed (in the sense of providing a detailed musical score) or have high degrees of indeterminacy where the performer determines the outcome. In Steve Reich’s phase-shifting music, we experience a kind of temporal rupture that comes about when a single, repeated musical sequence gradually becomes out of phase with itself. While initially we might be able to capture all of the sounds in a single auditory stream, at some point (due to the limits of our perceptual ordering faculties), the music suddenly appears to split into two or more auditory streams (Wilson 1999).

While composers of indeterminate and minimalist music have experimented with different forms of *non-linear* time (often drawing heavily on technology), even more traditional musical forms, such as the canon and fugue, provide some useful ways of thinking about how we design our educational *textures* and therefore, what opportunities texture might bring in terms of creating openings or lacunae. Hoftstadter (1979) likens Bach’s *Canon per Tonos* (from the *Musical Offering*), which he describes as an ‘endlessly rising canon’ with Godel’s notion of ‘strange loops’ and the paradoxes inherent in Escher’s drawings. Hoftstadter suggests that the strange loop emerges from a tangled hierarchy, and notes that the canon moves further and further away from its starting point but then suddenly it is back (Hoftstadter 1979: 10, 15). Canons were often not written out in full. It was common to ‘give a single theme, together with some more or less tricky hints and to let the canon based on that theme be ‘discovered’ by someone else’ (8).

The discussion above also prompts us to think about the notion of counterpoint (the relationship between two or more musical lines), and how the idea of counterpoint, which contributes to a musical texture, might translate in the context of education. The model proposed above highlighted concurrence as an important and perhaps underexplored element of education (in terms of the work of educational design). The learning environment is complex, and there are many ways we can try to shape it to achieve our educational goals. What kinds of approaches and environments might help us combine things in new ways that disrupt linear forms and create potential openings for new ideas, understandings or experiences to emerge; and what role might indeterminacy and improvisation plays in this process?

## Listening to the Post-script

We might consider that an important part of improvisation (and creativity in general) is recognising that the ‘afterthought’ or ‘postscript’ is often the thing that will lead us to interesting places. Sometimes the afterthought then becomes the central idea, and then we begin to improvise again. The notion of the post-script prompts another question here—what happens when we go beyond the script, and move to the other end of the continuum, towards randomness? Is part of our task, in realising the sonic possibilities in learning and teaching, to learn how to listen to the post-script, that is, to listen in a more deliberate way to the aspects of learning and teaching that aren’t tied to a script?

Biesta (2017) cautions against conceptions of education that relegate the teacher to the guide on the side and closely examines what we might be losing by taking this stance. He proposes a new role for the teacher, connected to the purpose of education, which he sees as helping students stay in the ‘middle ground’ where they are in dialogue ‘with the other’ in a grown-up way. He also proposes that the way time is understood in education—as growth, change and progress—is not always helpful. Rather he suggests that the temporal dimension in education should be non-temporal in order to support a kind of education focused on emancipation.

Perhaps there is another way of looking at this by examining the role of improvisation more closely; one where the guide on the side is not simply about setting active learning tasks for students to do, but going further than facilitation, into improvisational modes, that allow adjustments on the fly, and involve responsively shaping the ways in which students engage in temporal and non-temporal, linear and non-linear forms of experience which may either lead to learning, or serve to hold them in a space that allows them to examine the desirability of their desires. Perhaps the argument should be less focused on the dichotomy between transmission versus active learning, and more energy given to finding approaches that help us to recognise at what points these different modes might help students stay in the middle-ground, in dialogue with the other, as subject, and to be able to move fluently between them. This would require a more nuanced understanding of how people, tasks and technology come together in complex learning environments.

This paper began by discussing how current research describes the role of improvisation in education, and has argued for a broader consideration of the role of improvisation in education and its potential to positively disrupt traditional linear timescapes. The model adapted from Stern (1988) is intended to support educators in considering the role of improvisation in their design and teaching practice, ways in which technology might support or constrain improvisatory practices, and to highlight new themes such as silence, absence, openings, and rupture, that also have resonance in postdigital education. It is suggested that models of improvisation and indeterminacy in education may provide useful practical tools for educational designers and teachers, and that deeper explorations of linear and non-linear educational forms that draw on sound and music studies would have much to offer the field of education.

Improvisation relies on listening, responding, fluidity, and the ability to act in the moment. In collaborating with others in this mode, elements such as access, attention and proximity are important. These characteristics resonate with the qualities of relationship-rich education (Felton and Lambert 2021). We know from studies in education exploring teaching in the age of Covid-19 (Jandrić et al. 2020, 2021) that many teachers and students experienced significant challenges with aspects of communication and relationship building with the shift to online learning, but also that people were adapting, and technology evolving, to better support the kinds of educational experiences we value. Future research can help us better understand the role of technology and its interrelationship with other elements in supporting improvisational processes in education. In music, research has investigated criteria for facilitating collaboration in online group musical improvisations. The research focuses on aspects such as localisation, mutual awareness, mutual modifiability, signalling group configurations, sound visualisations and shared and consistent representations (Cai and Terry 2020). Similar advances in education would help us to move more fluently between educational environments, and not be ‘over attached, or invested into, one particular medium’ (Cramer and Jandrić 2021).

Prevost (1982) suggests that ‘forms of music reflect general ideas of social and individual aspiration’ and contemporary improvised music reflects ‘legitimate aspirations which arise as a specific and revolutionary response to the world as we find it now’ (37). The challenge to improvisation in Western art music appeared gradually, yet increasingly, coinciding with the development of Western industrial society which began to favour the compositional mode. As suggested by Durant (1989), this transition took place when ‘shared patterns of formal repetition and permutation [were] replaced by a far more fully calculated uniqueness of formal development’ (215). Provost reminds us that:

Contrary to the fear of those who claim to defend our music tradition from degeneration, improvisation is not a threat to the integrity of music. Historically the reverse is palpably true … Improvisation has, of course, withered away from Western ‘serious’ music almost to the point of extinction. But this trend is counter to tradition itself. (Durant 1989: 216)

In line with this, Durant (1989) notes that it is precisely the relations between music and other levels of societal formations which improvisation, perhaps more than any other aspect of music making today, investigates and disrupts (217). I argue that not only should we consider how improvisation and indeterminacy might be used to challenge traditional timescapes in education, but we should also consider the actual sonic environments that these forms generate; thinking about the distinctions between sound and noise, noting that noise and sound are social conceptions (Ford and Sasaki 2021), and bringing these conversations into our design processes and into the learning environment with our students. Further thought needs to be given to how design that pays more deliberate attention to designing for indeterminacy might support an orientation towards the unforeseen—to what is not present, and to borrow Cascone’s term, to helping educational designers, teachers, and students to find their ‘inner constellation’ (Cascone and Jandrić 2021: 571).

## Data Availability

Not applicable.
